# Apocrine Adenoma of the External Auditory Canal with Pseudoepitheliomatous Hyperplasia

**DOI:** 10.1155/2019/7395856

**Published:** 2019-02-20

**Authors:** Masafumi Ohki, Shigeru Kikuchi

**Affiliations:** Department of Otolaryngology, Saitama Medical Center, Saitama Medical University, 1981 Kamoda, Kawagoe-shi, Saitama 350-8550, Japan

## Abstract

The tumors derived of the ceruminous gland in the external auditory canal are rare. Here, we report a case of a ceruminous adenoma (apocrine adenoma) with refractory chronic inflammation in the external auditory canal. A 46-year-old man presented with otorrhea, itching, and a foreign body sensation in his right ear. A soft reddish protruding lesion was revealed at the posterosuperior portion of the entry to the right external auditory canal by otoscopy. The skin lesion was endaurally resected; histopathology showed luminal structures in the middle to deep layer of the epidermis and inflammatory granulation below pseudoepitheliomatous hyperplasia. The walls of the luminal structures consisted of inner luminal secretory cells featuring apical decapitation secretion and outer myoepithelial cells. The patient was diagnosed with an apocrine adenoma. Three years after surgery, there has been no evidence of recurrence. Complete resection, including the deep layer of the epidermis, is necessary.

## 1. Introduction

Ceruminous gland tumors of the external auditory canal are rare and are derived from sweat gland tumors in the external auditory canal. Sweat glands are classified as eccrine or apocrine glands. Eccrine sweat glands are absent from the external auditory canal, whereas apocrine sweat glands are distributed in the outer one-third of the external auditory canal [1]. Therefore, the ceruminous gland tumors of the external auditory canal generally originate from apocrine sweat glands. Here, we report a case of a ceruminous adenoma (apocrine adenoma) in the external auditory canal accompanied by refractory chronic inflammation.

## 2. Case Presentation

**Figure 1:**
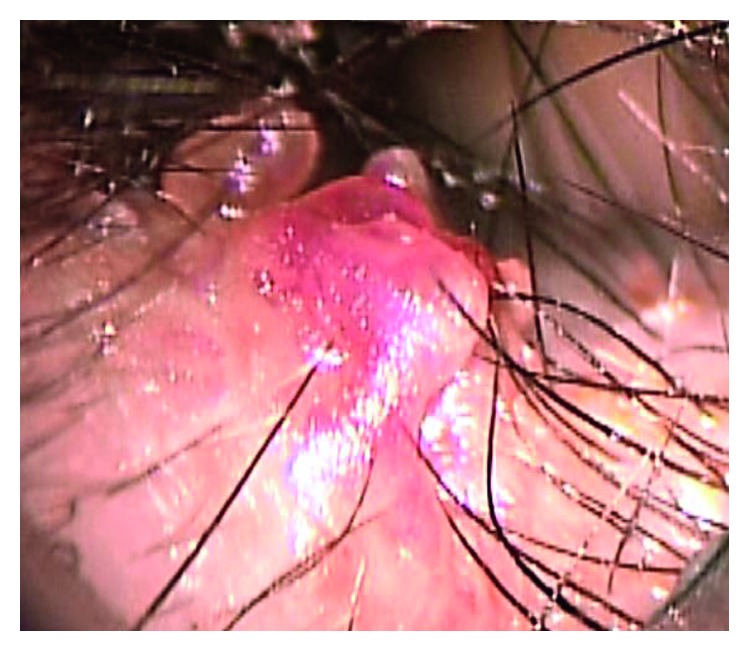
Gross appearance of the tumor. Photograph showing a soft reddish protruding lesion at the entry of the external auditory canal.

**Figure 2:**
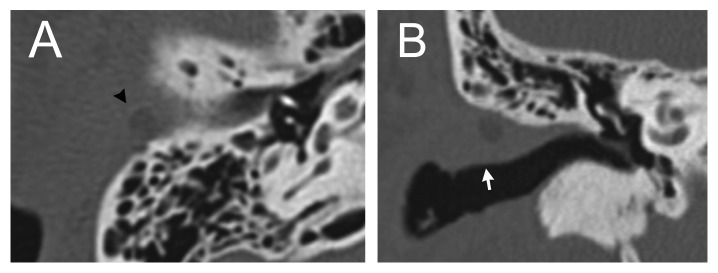
Computed tomography of the temporal bone. The axial view (A) and coronal view (B) demonstrate a dense, protruding, soft tissue lesion overlying the cartilage in the posterosuperior portion of the entry of the external auditory canal, without mastoid or middle ear lesions.

**Figure 3:**
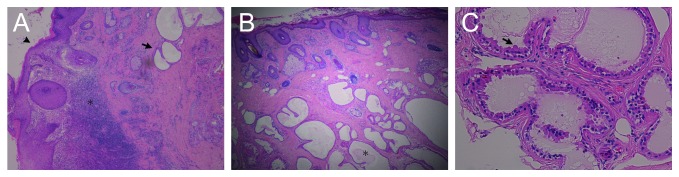
Histopathology of the tumor. Luminal structures (arrow) and inflammatory granulation (asterisk) below pseudoepitheliomatous hyperplasia (arrow head) (hemotoxylin and eosin (HE) stain, ×40) (A). Luminal structures (asterisk) containing eosinophilic cytoplasm proliferated in the middle to the deep layer of the epidermis (HE staining, ×40) (B). The luminal structures were composed of dual cell layers consisting of inner luminal secretory cells with apical decapitation secretion (arrow) and outer myoepithelial cells (HE staining, ×200) (C).

A 46-year-old man presented with otorrhea, itching, and a foreign body sensation in his right ear. Otoscopic examination revealed a soft reddish protruding lesion at the posterosuperior portion of the entry to the right external auditory canal (Figure 1). Computed tomography of the temporal bone showed a dense, protruding soft tissue lesion of the skin overlying the cartilage of the external auditory canal, but there was no evidence of the mastoid or middle ear lesions (Figure 2). A tissue biopsy showed granulation tissue. Treatment with ointment containing gentamicin sulfate was ineffective. The skin lesion was endaurally resected; pathologic examination showed luminal structures in the middle to deep layer of the epidermis and inflammatory granulation below pseudoepitheliomatous hyperplasia (Figures 3(a) and 3(b)). The walls of the luminal structures consisted of inner luminal secretory cells and outer myoepithelial cells (Figure 3(c)). These inner luminal secretory cells showed apical decapitation secretion.

The patient was diagnosed with an apocrine adenoma and pseudoepitheliomatous hyperplasia with inflammatory granulation. After surgery, otorrhea due to slight inflammatory granulation was prolonged. Therefore, additional resection of the posterosuperior portion of the ear auditory canal entry, including the cartilage over the bone, was conducted. The skin defect of the posterosuperior portion of the auditory canal was reconstructed using a postauricular island pedicle skin flap. The otorrhea and inflammation resolved. Three years after surgery, there has been no evidence of recurrence.

## 3. Discussion

The most characteristic macroscopic feature of a ceruminous adenoma is a polypoid mass with a reddish to reddish brown appearance, and most cases show surface ulceration [1]. Asymptomatic cases are common, but a few patients present with canal obstruction and hearing loss by the mass; very rare symptoms include facial paralysis, otorrhea, otalgia, and bleeding [1]. Reported ceruminous adenomas in the external auditory canal originate from apocrine glands; only two cases of eccrine adenoma have been reported [2, 3].

A difference between eccrine and apocrine sweat glands is that apocrine glands consist of columnar cells containing eosinophilic cytoplasm with apical decapitation secretion, whereas eccrine glands do not demonstrate apical decapitation secretion [1, 4]. In 1971, Wetli et al. first classified the neoplasms originating from ceruminous glands of the external auditory canal as adenomas, adenocarcinomas, adenoid-cystic carcinomas, and mixed tumors (pleomorphic adenomas) [5]. In 1992, Mansour et al. reappraised the classification as adenoma, pleomorphic adenoma, syringocystadenoma papilliferum, benign eccrine cylindroma, adenoid-cystic carcinoma, adenocarcinoma, and mucoepidermoid carcinoma [6]. The most recent classification in 2004 by Thompson et al. includes benign neoplasms such as ceruminous adenomas, ceruminous pleomorphic adenomas, and ceruminous syringocystadenoma papilliferum and malignant neoplasms such as ceruminous adenocarcinomas, ceruminous adenoid-cystic carcinomas, and ceruminous mucoepidermoid carcinomas [1].

The features of ceruminous neoplasms derived from apocrine glands include existence of the walls of the luminal structures consisting of inner luminal secretory cells and outer myoepithelial cells. The apical decapitation secretion of inner luminal secretory cells strongly suggests origination from the apocrine glands [1]. The differential features of a ceruminous pleomorphic adenoma and a syringocystadenoma papilliferum from a ceruminous adenoma include the presence of the myxoid-chondroid matrix material and papillary projections into a cystic lumen, respectively [1]. Ceruminous adenocarcinomas, ceruminous adenoid-cystic carcinomas, and ceruminous mucoepidermoid carcinomas are malignant tumors and are different from benign ceruminous adenoma tumors.

The pathology of our case shows accumulation of inflammatory cells below the pseudoepitheliomatous hyperplasia. The inflammation probably caused otorrhea and itching. The preoperative tissue biopsy did not demonstrate an apocrine adenoma, but granulation as the tumor was not located in the deep layer of the epidermis at the biopsy site.

Treatment for an apocrine adenoma is complete surgical resection. The ceruminous glands are placed in the deep layer of the dermis close to the cartilage [7]. Therefore, the subcutaneous tissues above the surface of the cartilage and epidermis that include the tumor should be completely excised; otherwise, recurrence is inevitable. Our case required additional wide resection that included the cartilage.

Apocrine adenomas of the external auditory canal occur in the deep layer of the epidermis; there is inadequate tissue biopsy for diagnoses, and wide resection, including the deep layers of the epidermis, is required. Otherwise, recurrence is inevitable.

Apocrine adenomas should be treated as potentially malignant. Apocrine adenomas are difficult to distinguish from well-differentiated apocrine adenocarcinomas, as the presence or absence of invasion into the tumor margin is the differentiating feature; the high incidence of malignancy has been pointed out as well [6].
